# Isolated Aphasia as an Epileptic Manifestation of Neurosarcoidosis: A Case Report

**DOI:** 10.7759/cureus.115786

**Published:** 2026-09-04

**Authors:** Martin Caluwaerts, Benjamin Legros, Valentine Melas, Dylan Mommens, Yahya Lablad

**Affiliations:** 1 Emergency Medicine, Hôpital Universitaire de Bruxelles (HUB), Erasme Hospital, Brussels, BEL; 2 Neurology, Hôpital Universitaire de Bruxelles (HUB), Erasme Hospital, Brussels, BEL; 3 Family Medicine, Université Catholique de Louvain (UCLouvain), Brussels, BEL; 4 Radiology, Hôpital Universitaire de Bruxelles (HUB), Erasme Hospital, Brussels, BEL; 5 Internal Medicine, Hôpital Universitaire de Bruxelles (HUB), Erasme Hospital, Brussels, BEL

**Keywords:** emergency medicine, epileptic aphasia, focal epilepsy, hydrocephalus, neurological emergency, neurosarcoidosis, nonconvulsive status epilepticus, sarcoidosis, stroke mimic

## Abstract

Acute aphasia requires urgent evaluation for stroke, but recurrent and stereotyped language disturbances may also reflect focal seizures.

We report the case of a 23-year-old man brought to the emergency department (ED) with aphasia that had been present for approximately six hours. Over the preceding week, he had experienced several similar but briefer episodes that had resolved spontaneously. He was concurrently undergoing investigation at another institution for persistent hiccups and near-daily vomiting of more than one month’s duration. Chest computed tomography (CT) and positron emission tomography (PET)-computed tomography had already shown mediastinal and mesenteric lymphadenopathy suggestive of sarcoidosis, although other etiologies, including malignancy, could not be excluded.

Neurological examination was limited by the language disturbance but showed no obvious motor deficit. Brain computed tomography revealed obstructive hydrocephalus with transependymal cerebrospinal fluid flow and multiple enhancing nodular lesions, predominantly in and around the ventricular system. Electroencephalography demonstrated epileptiform activity, and the aphasia resolved after treatment with levetiracetam and clobazam. Further cerebrospinal fluid analysis, magnetic resonance imaging (MRI), and pathological investigations supported a diagnosis of probable neurosarcoidosis presenting with lymphocytic meningoencephalitis and obstructive hydrocephalus.

This case illustrates an unusual pathophysiological sequence, isolated aphasia as a focal epileptic manifestation of neurosarcoidosis, and is a reminder that unexplained focal neurological symptoms, particularly when fluctuating or recurrent, warrant the consideration of seizures and early electroencephalography.

## Introduction

Aphasia is an acquired language disorder characterized by the impairment of language production, comprehension, or both [1]. Its overall incidence is difficult to determine because aphasia has multiple etiologies and transient cases may not be captured. Estimates based largely on stroke data range from approximately 20 to 60 per 100,000 person-years in developed countries [2].

In the emergency department (ED), aphasia may be difficult to distinguish from delirium, dysarthria, psychiatric disorders, apraxia of speech, and other neurological conditions. This distinction is clinically important because it directs the etiological workup and determines management. Stroke is the leading cause of aphasia and must be excluded urgently because of the narrow therapeutic window [3]. When aphasia is isolated, however, cerebral ischemia is less frequent, although it remains an important possibility [4,5]. Focal seizures should also be considered, as isolated aphasia is an uncommon but recognized epileptic manifestation [6-8].

Sarcoidosis is an uncommon systemic granulomatous disorder with a reported prevalence ranging from approximately 1-5 to more than 100 per 100,000 across populations [9]. Symptomatic neurological involvement occurs in about 5% of patients, although subclinical nervous system involvement may be more frequent [10,11]. Neurosarcoidosis can present with meningitis, parenchymal lesions, hydrocephalus, and seizures [10-12]. Although language disturbances and seizures have each been reported, isolated aphasia associated with documented epileptiform activity appears to be exceptionally rare in patients with sarcoidosis [13,14].

We report a 23-year-old man whose recurrent isolated aphasia was attributed to focal epileptic activity in the setting of probable neurosarcoidosis with lymphocytic meningoencephalitis and obstructive hydrocephalus. The case illustrates the convergence of several uncommon events: symptomatic neurosarcoidosis with lymphocytic meningoencephalitis and obstructive hydrocephalus, focal epileptic activity, and isolated aphasia as the sole clinically apparent ictal manifestation. This sequence created a particularly challenging stroke mimic in the emergency setting.

## Case presentation

A 23-year-old man was brought by ambulance to the ED during the night because of aphasia that had persisted for approximately six hours. His father reported several similar but shorter episodes during the preceding week, each of which had resolved spontaneously. The prolonged duration of the current episode prompted the emergency call. Because of the language disturbance, the history was obtained mainly from his father. There was no reported fever, headache, loss of consciousness, or focal motor symptoms. One episode of vomiting had occurred approximately one hour before the onset of aphasia.

For more than one month, the patient had been undergoing investigation at another hospital for persistent hiccups and almost daily vomiting. According to the available radiology reports, chest computed tomography (CT) and positron emission tomography-computed tomography (PET-CT) had shown mediastinal and mesenteric lymphadenopathy, raising suspicion for sarcoidosis, although other etiologies, including malignancy, could not be excluded. Needle aspiration of a mediastinal lymph node had been performed, but the results were not yet available. His medications included baclofen for hiccups, domperidone as needed for vomiting, and pantoprazole for erosive gastropathy.

On arrival, the heart rate was 89 beats/minute, blood pressure was 123/82 mmHg, respiratory rate was 18 breaths/minute, oxygen saturation was 99% on room air, temperature was 36.6°C, and capillary blood glucose was 100 mg/dL. The patient had marked aphasia. He appeared to understand some simple questions or commands but responded almost exclusively with yes or no and could not reliably complete formal language or motor testing. He moved all four limbs spontaneously and symmetrically, without an obvious focal motor deficit. Mild neck stiffness was present. Deep tendon reflexes were symmetric, plantar responses were flexor bilaterally, and the Hoffmann sign was mildly positive on the left. The remainder of the general examination was unremarkable.

Electrocardiography was normal. Laboratory testing was unremarkable except for neutrophilia of 12,450 cells/mm³. Emergency brain CT, including noncontrast, arterial, and delayed post-contrast acquisitions, showed enlargement of the lateral ventricles with periventricular hypoattenuation consistent with transependymal cerebrospinal fluid flow. Multiple enhancing nodular lesions were present in the supra- and infratentorial compartments, predominantly in and around the ventricular system. Several lesions involved the third ventricle and the foramina of Monro, accounting for obstructive hydrocephalus. The initial radiological differential diagnosis included infection, metastatic disease, and neurosarcoidosis (Figure 1, left column).

**Figure 1:**
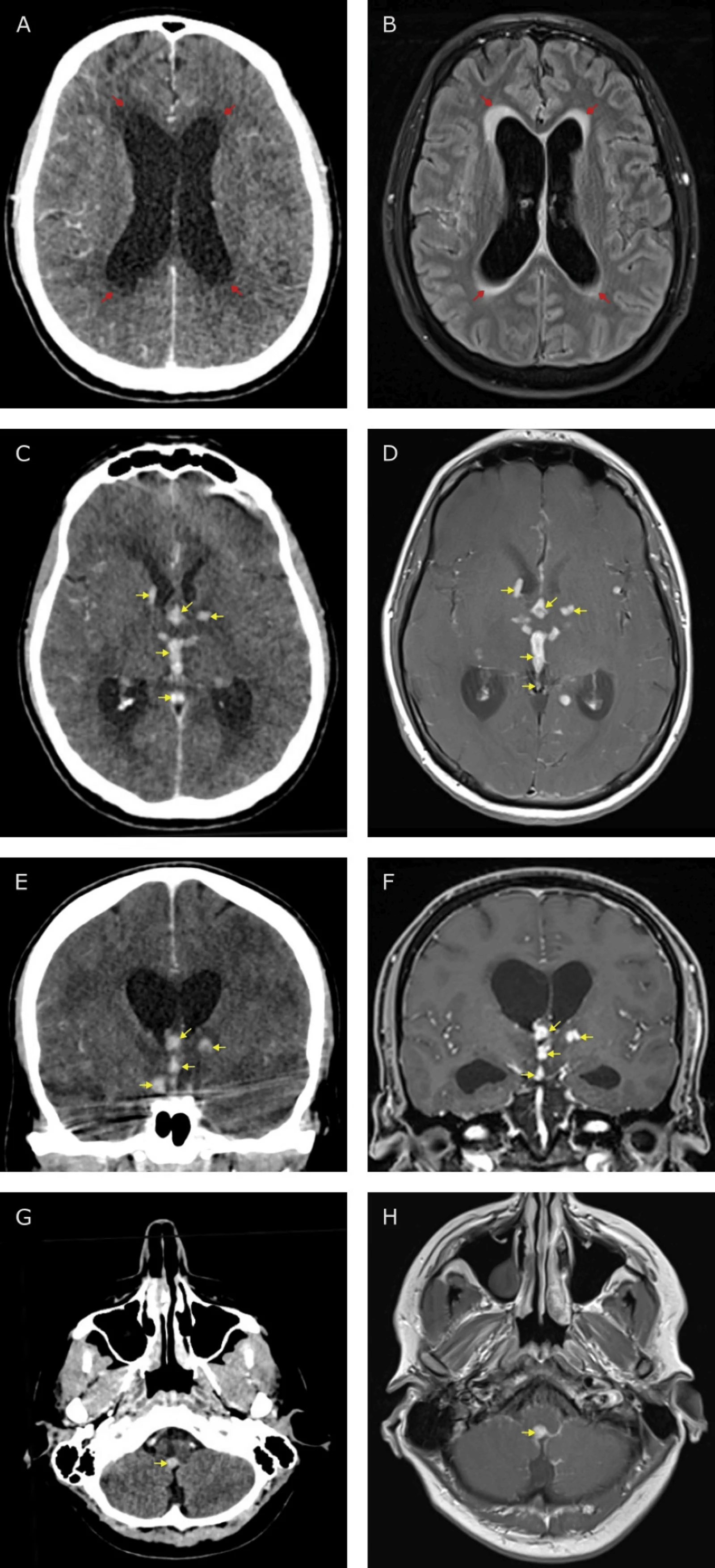
Corresponding computed tomography (left column) and magnetic resonance imaging (right column) findings demonstrating multifocal central nervous system involvement and obstructive hydrocephalus. (A and B) Axial contrast-enhanced CT and axial FLAIR MRI demonstrating dilatation of the lateral ventricles with periventricular signal abnormality (red arrows) consistent with transependymal cerebrospinal fluid flow. (C and D) Axial contrast-enhanced CT and post-contrast T1-weighted MRI demonstrating multiple enhancing nodular lesions (yellow arrows) in and around the third ventricle. (E and F) Coronal contrast-enhanced CT and post-contrast T1-weighted MRI demonstrating enhancing lesions (yellow arrows) involving the third ventricle and foramina of Monro, contributing to obstructive hydrocephalus. (G and H) Axial contrast-enhanced CT and post-contrast T1-weighted MRI demonstrating an enhancing lesion (yellow arrow) in the medulla adjacent to the fourth ventricle. CT, computed tomography; FLAIR, fluid-attenuated inversion recovery; MRI, magnetic resonance imaging

The patient was transferred to the hospital where his systemic investigation was already in progress. Electroencephalography was performed while the aphasia was ongoing and showed left lateralized rhythmic delta activity (LRDA) with left frontal epileptiform discharges (Figure 2). On the basis of these findings, levetiracetam was initiated, and clobazam was administered. The aphasia subsequently resolved. The recurrent stereotyped episodes, electroencephalographic abnormalities recorded during the deficit, and temporal response to antiseizure treatment strongly supported an epileptic origin of the language disturbance.

**Figure 2:**
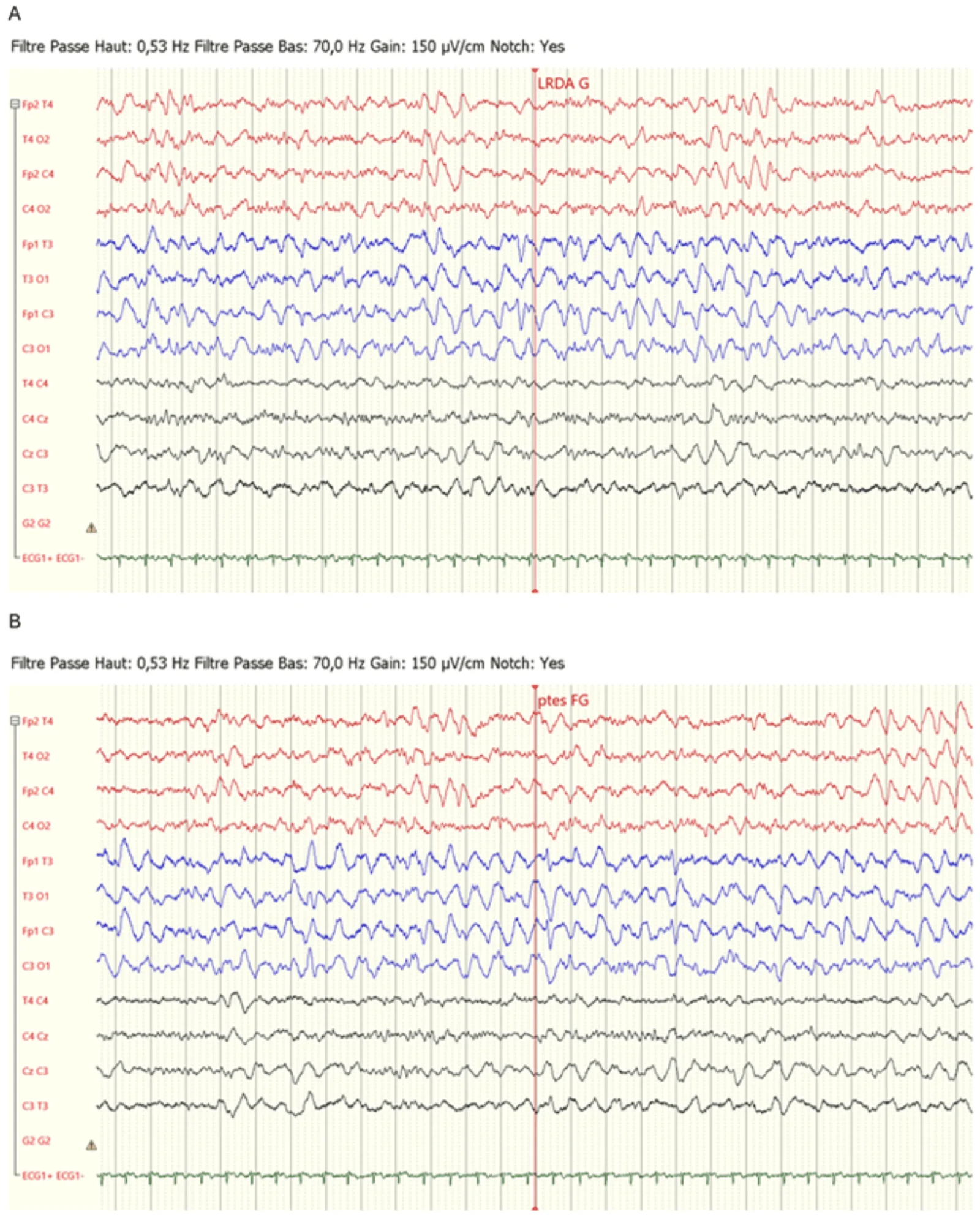
Electroencephalographic abnormalities recorded while the patient remained aphasic. Representative 25-second EEG epochs were recorded using the international 10-20 electrode system and displayed in a longitudinal bipolar montage (high-pass filter, 0.53 Hz; low-pass filter, 70 Hz; notch filter on; sensitivity, 150 μV/cm). (A) Left lateralized rhythmic delta activity (LRDA), maximal over the left frontotemporal derivations. (B) Left frontal epileptiform discharges superimposed on the lateralized rhythmic slowing.

Further evaluation included cerebrospinal fluid analysis, contrast-enhanced brain magnetic resonance imaging (MRI), an extensive infectious and inflammatory workup, and repeat mediastinal lymph node sampling. Cerebrospinal fluid showed a lymphocytic inflammatory profile, while magnetic resonance imaging confirmed multifocal central nervous system involvement and obstructive hydrocephalus (Figure 1, right column). Pathological examination of the lymph node specimen demonstrated findings compatible with systemic sarcoidosis, with no evidence of infection or malignancy. Together, these findings fulfilled the Neurosarcoidosis Consortium Consensus Group criteria for probable neurosarcoidosis, presenting with lymphocytic meningoencephalitis and obstructive hydrocephalus [11].

Initial treatment with methylprednisolone 64 mg daily did not produce sufficient improvement. Treatment was escalated to pulse corticosteroid therapy, methotrexate with folic acid, and infliximab (5 mg/kg intravenously at weeks 0, 2, and 6 and then every eight weeks). The hydrocephalus required ventriculoperitoneal shunt placement.

The subsequent course was complicated by postoperative *Acinetobacter ursingii* meningitis, which required 17 days of meropenem and temporary external ventricular drainage before a ventriculoperitoneal shunt was reinserted. His erosive gastropathy worsened during corticosteroid treatment and was complicated by hematemesis.

After approximately two months of acute hospitalization, he was transferred to inpatient rehabilitation. The Functional Independence Measure (FIM) score improved from 104/126 (cognitive, 21/35; motor, 83/91) at rehabilitation admission to 124/126 (cognitive, 33/35; motor, 91/91) after approximately seven weeks.

At six months of follow-up, treatment still included levetiracetam, methotrexate, methylprednisolone, and maintenance infliximab every eight weeks. No recurrent seizures or aphasic episodes were reported.

## Discussion

Isolated aphasia in the emergency department: Looking beyond stroke

Stroke appropriately remained the first diagnosis to exclude. Isolated aphasia may occur in ischemic stroke or transient ischemic attack, and transient isolated aphasia may be associated with a cardioembolic source [4,5]. At the same time, isolated language disturbance has a broad differential diagnosis, and seizures are an important stroke mimic [4,6]. Several features in this patient argued against a typical vascular presentation without eliminating it: he was young, had no obvious motor deficit, and had experienced multiple stereotyped, self-limited episodes during the preceding week.

Toxic-metabolic and psychiatric causes were also considered. A psychiatric disorder remained a diagnosis of exclusion in a patient without a relevant psychiatric history, while toxicological screening would have been reasonable if structural imaging had not provided an explanation. The multiple enhancing intracranial lesions and obstructive hydrocephalus instead established that the presentation occurred in the context of severe structural and inflammatory central nervous system disease.

Evidence for an epileptic mechanism

Isolated aphasia is a rare manifestation of focal seizures and can be difficult to recognize in the absence of convulsive activity or an obvious alteration of consciousness [7,8]. This difficulty is reflected in the substantially longer diagnostic delay reported for focal nonmotor seizures compared to focal motor seizures [7]. In retrospect, the brief, recurrent, and stereotyped aphasic episodes during the preceding week were an important clue.

The epileptic origin of the aphasia was supported by several concordant findings: recurrent stereotyped episodes during the preceding week, left lateralized rhythmic delta activity (LRDA) with superimposed left frontal epileptiform discharges recorded while the patient remained aphasic, and the resolution of the language deficit after levetiracetam and clobazam were initiated.

LRDA is a standardized electroencephalographic pattern [15]. In critically ill cohorts, it has been associated with a high risk of acute, predominantly nonconvulsive seizures [16]. In the present case, the occurrence of this pattern during the language deficit, the superimposed epileptiform discharges, and the subsequent therapeutic response provided strong electroclinical support for the diagnosis of an aphasic seizure.

Aphasia and seizures in neurosarcoidosis

Published reports of aphasia in neurosarcoidosis are scarce. Duffey and Bates described recurrent abrupt nonfluent dysphasia in a patient with sarcoidosis, but no epileptic activity or macroscopic granulomatous lesion was demonstrated [14]. In a later series of nine patients, Shrestha et al. described an adolescent with reversible aphasia accompanied by unilateral facial weakness; an epileptic mechanism was not documented [13]. Neither report therefore described the combination of isolated aphasia, focal epileptiform activity recorded during the deficit, and clinical resolution after antiseizure treatment observed in the present case.

Seizures are a recognized manifestation of neurosarcoidosis and tend to occur in patients with more extensive central nervous system involvement. In the historical cohort of Krumholz et al., 13 of 79 patients (16%) had seizures; 12 of the 13 patients (92%) experienced generalized tonic-clonic seizures, while four also had partial seizures [17]. Seizures were associated with intracranial mass lesions, encephalopathy or vasculopathy, and hydrocephalus rather than acting as an isolated prognostic factor [17]. A recent French multicenter cohort reported seizures in approximately one-fifth of patients with central nervous system neurosarcoidosis [18].

In this case, the rarity lies not in any single manifestation but in the sequence linking them. A systemic granulomatous disease produced multifocal central nervous system involvement, including lymphocytic meningoencephalitis and intraventricular lesions causing obstructive hydrocephalus. Within this severe structural and inflammatory context, the patient developed a left hemispheric focal epileptic process that presented clinically as isolated aphasia, without convulsions, loss of consciousness, or an obvious motor deficit. The combination of an uncommon neurological disease, an uncommon complication, and an uncommon nonmotor ictal presentation created a particularly deceptive emergency presentation.

Hydrocephalus and disease severity

Hydrocephalus is an uncommon but severe manifestation of neurosarcoidosis. Published cohorts and reviews generally place its frequency in the single-digit percentage range [12,19]. It is often obstructive and may result from granulomatous meningitis, intraventricular inflammation, or focal obstruction of cerebrospinal fluid pathways. In the cohort of ten Dam et al., obstruction was located at the foramina of Monro in three of 10 patients with an identified obstruction site (30%), matching the anatomical mechanism seen in our patient [19].

A review of 36 published cases found a mortality of 22%, although the authors noted that publication bias may have inflated this estimate; mortality in their own contemporary cohort was 9% [19]. Older reports described still higher mortality when hydrocephalus coexisted with seizures, but these estimates arose from small and highly selected historical populations and should not be applied directly to current patients [17,19]. The coexistence of seizures, multifocal lesions, meningoencephalitis, and obstructive hydrocephalus nevertheless reflected severe central nervous system involvement and was consistent with the need for both cerebrospinal fluid diversion and escalating immunosuppression.

Establishing the diagnosis

Importantly, the term probable reflects the formal Neurosarcoidosis Consortium Consensus Group classification rather than substantial uncertainty regarding the diagnosis. Under these criteria, a compatible neurological syndrome with objective evidence of nervous system inflammation and histologically confirmed extraneural sarcoidosis, after the reasonable exclusion of alternative diagnoses, is classified as probable neurosarcoidosis; definite neurosarcoidosis requires histological confirmation from neural tissue [11]. In our patient, lymphocytic meningoencephalitis, multifocal enhancing central nervous system lesions, and obstructive hydrocephalus established a compatible neurological syndrome, while mediastinal lymph node histopathology confirmed systemic sarcoidosis. Infectious and malignant causes, which had been considered on the initial imaging, were not supported by the subsequent evaluation. The case was therefore appropriately classified as probable neurosarcoidosis.

Treatment and clinical implications

Evidence guiding the treatment of neurosarcoidosis remains largely observational. Corticosteroids are generally used initially, while steroid-sparing immunosuppressive therapy and tumor necrosis factor inhibition may be required in severe or refractory disease [20]. Hydrocephalus often requires both immunosuppression and neurosurgical cerebrospinal fluid diversion [19,20]. The prolonged hospitalization, shunt-related infection, gastrointestinal bleeding, and need for rehabilitation in this patient illustrate how morbidity may arise from the neurological disease, neurosurgical treatment, and immunosuppressive therapy.

The main emergency medicine lesson is not to replace urgent stroke evaluation with an epilepsy-first approach but to broaden the differential diagnosis when isolated aphasia is recurrent, fluctuating, or unexplained by vascular imaging. Electroencephalography should be obtained early when possible. In this patient, urgent imaging revealed severe structural disease, while EEG clarified the immediate mechanism of the language deficit and directed effective symptomatic treatment.

Limitations

This report has several limitations. The precise aphasic syndrome could not be characterized because the severity of the language disturbance prevented the reliable assessment of repetition and other language domains. In addition, the temporal association between antiseizure treatment and clinical recovery, although compelling, cannot independently establish causality in a single observation.

## Conclusions

This case documents an unusual electroclinical sequence in which probable neurosarcoidosis with lymphocytic meningoencephalitis, multifocal central nervous system lesions, and obstructive hydrocephalus was complicated by focal epileptic activity presenting solely as aphasia. The recurrent stereotyped episodes, left-sided epileptiform abnormalities recorded while the patient remained aphasic, and subsequent resolution after levetiracetam and clobazam strongly supported an epileptic mechanism. Although acute isolated aphasia should continue to prompt urgent evaluation for stroke, a recurrent or fluctuating pattern, particularly in the presence of structural or inflammatory central nervous system disease, should lead to early electroencephalography and the consideration of focal seizures. This case broadens the recognized clinical spectrum of neurosarcoidosis and illustrates how a systemic inflammatory disease with severe central nervous system involvement may initially present with a deceptively focal, nonmotor symptom.
